# Differential fibril morphologies and thermostability determine functional roles of *Staphylococcus aureus* PSMα1 and PSMα3

**DOI:** 10.3389/fmolb.2023.1184785

**Published:** 2023-07-04

**Authors:** Bader Rayan, Eilon Barnea, Alexander Khokhlov, Alexander Upcher, Meytal Landau

**Affiliations:** ^1^ Department of Biology, Technion-Israel Institute of Technology, Haifa, Israel; ^2^ Ilse Katz Institute for Nanoscale Science and Technology, Ben Gurion University of the Negev, Beer-Sheva, Israel; ^3^ Centre for Structural Systems Biology (CSSB), Deutsches Elektronen-Synchrotron DESY, Hamburg, Germany; ^4^ The Center for Experimental Medicine, Universitätsklinikum Hamburg-Eppendorf (UKE), Hamburg, Germany; ^5^ European Molecular Biology Laboratory (EMBL), Hamburg, Germany

**Keywords:** amyloid, thermostabilily, virulence factor, cytotoxicity, PSMs, fibrillation

## Abstract

Phenol-soluble modulins (PSMs) are virulent peptides secreted by staphylococci that undergo self-assembly into amyloid fibrils. This study focuses on *Staphylococcus aureus* PSMα1 and PSMα3, which share homologous sequences but exhibit distinct amyloid fibril structures. Upon subjecting PSMα1 to an 80°C heat shock, it fibrillates into cross-β structures, resulting in the loss of cytotoxic activity. Conversely, PSMα3 cross-α fibrils undergo reversible disaggregation upon heat shock, leading to the recovery of cytotoxicity. The differential thermostability probably arises from the presence of hydrogen bonds along the β-strands within the β-sheets of the cross-β fibrils. We propose that the breakdown of PSMα3 fibrils into soluble species, potentially co-aggregating with membrane lipids, is crucial for its toxic process and enables the reversible modulation of its biological activity under stress conditions. In contrast, the formation of robust and irreversible cross-β fibrils by PSMα1 corresponds to its role in biofilm stability. These findings emphasize how the unique fibril morphologies and thermostability of PSMα1 and PSMα3 shape their functional roles in various environments of *S. aureus*.

## Introduction

Phenol-soluble modulins (PSMs) are a group of peptides that are secreted by staphylococci. Within *Staphylococcus aureus*, the PSM group consists of several peptides, including PSMα1-α4, N-AgrD (∼20 residues), PSMβ1-β2 (∼40 residues), and δ-toxin, which are encoded in the genome, and the cassette-encoded PSM-mec (McKevitt et al., 1990; Mehlin et al., 1999; Queck et al., 2009; Schwartz et al., 2014). PSMs play a crucial role as virulence factors, especially in highly pathogenic strains that frequently cause severe infections, posing a significant public health concern. These peptides exhibit various pathogenic mechanisms, including the lysis of human cells such as leukocytes and erythrocytes, the induction of inflammatory responses, and the facilitation of biofilm development, both in terms of formation and dispersal (Le et al., 2014).

The functional properties of PSMs are influenced by their expression levels and the surrounding environment, leading to functional diversity likely dictated by different conformational states. Notably, PSMs have been observed to form amyloid fibrils, which have been investigated in multiple studies (Schwartz et al., 2012; 2014; Marinelli et al., 2016; Tayeb-Fligelman et al., 2017; Salinas et al., 2018; Najarzadeh et al., 2021; Zaman and Andreasen, 2021; Zhou et al., 2021; Grando et al., 2022; Kreutzberger et al., 2022; Wang et al., 2023). Amyloids are typically associated with fatal neurodegenerative and systemic diseases. However, it is important to note that many organisms across different kingdoms of life produce and secrete amyloids through dedicated pathways to fulfill various physiological functions. These functions include the storage of peptide hormones, memory formation, regulation of transcription and translation, and acting as virulence factors in microbial pathogens (Pham et al., 2014; Otzen and Riek, 2019).

**FIGURE 1:**
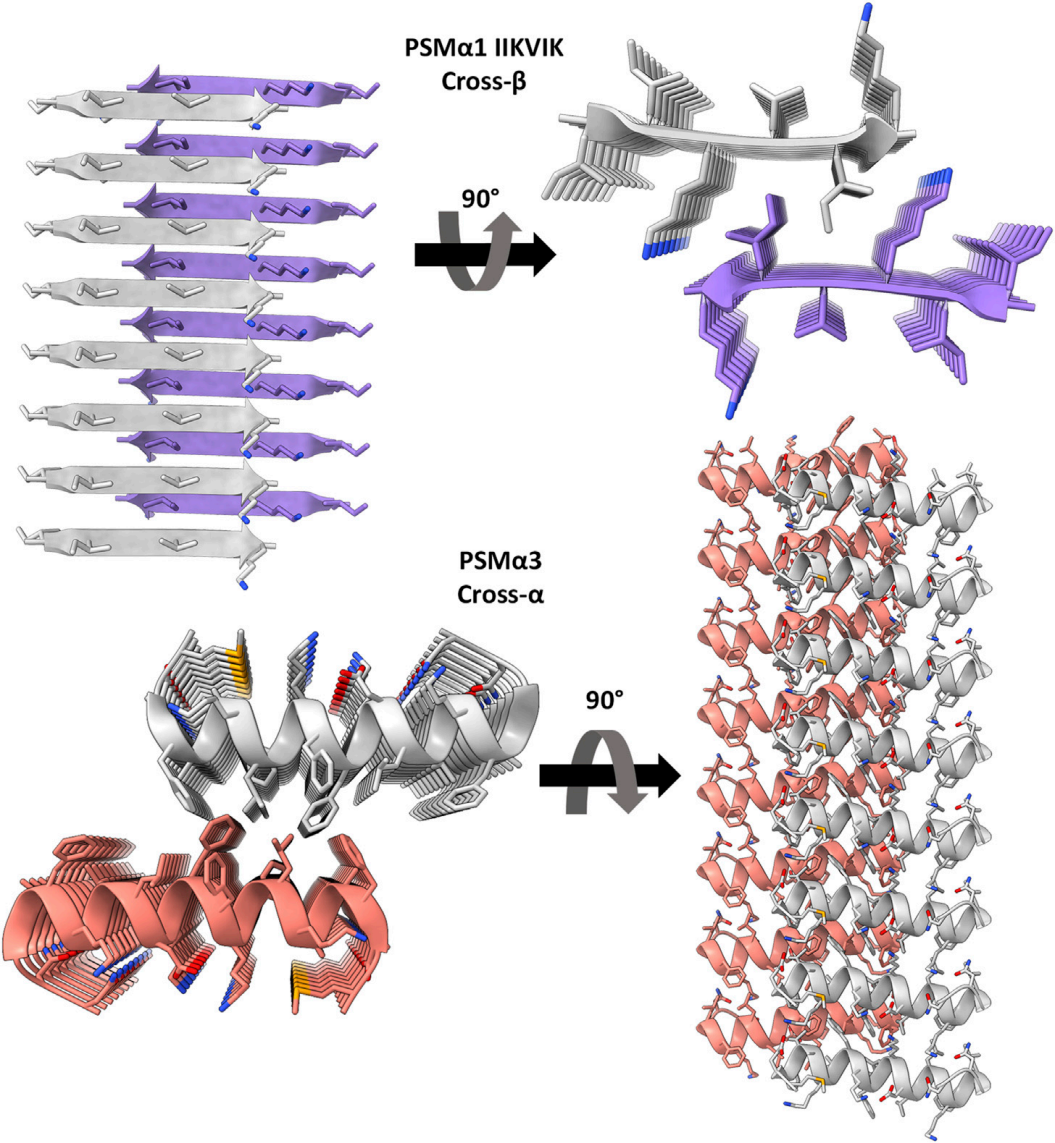
Crystal structures of PSMα3 and the spine segment of PSMα1. The crystal structure of PSMα1-IIKVIK (PDB id 6FG4) (Salinas et al., 2018) reveals a “steric-zipper” architecture, with tightly mated β-sheets composed of parallel β-strands. PSMα3 (PDB id 5I55) (Tayeb-Fligelman et al., 2017) exhibits a cross-α fibril structure composed of amphipathic α-helices that stack perpendicular to the fibril axis and form mated sheets. The depicted structures represent only eight layers of the fibrils, although actual fibrils contain thousands of layers. The left and right panels provide a view along and into the fibril axis, respectively (not drawn to scale), showcasing horizontally running β-strands or α-helices. The strands are shown as ribbons and residues as sticks. Each sheet and backbone atoms are colored differently; heteroatoms are colored according to their atom type (nitrogen in blue, oxygen in red).

Distinct from other protein fibrils, amyloids exhibit a unique self-assembly pattern, forming ordered fibrils composed of subunits arranged in sheets perpendicular to the fibril axis. These sheets are often comprised of β-strands, giving rise to the cross-β configuration (Nelson et al., 2005; Jahn et al., 2010). Surprisingly, the crystal structure of *S. aureus* PSMα3 unveiled a novel amyloid form termed cross-α, characterized by amphipathic α-helices stacked in paired sheets, reminiscent of the cross-β arrangement (Figure 1), and exhibiting amyloid dye binding. Recent cryo-electron microscopy studies confirmed the presence of the cross-α configuration in PSMα3, revealing a supramolecular assembly of mated α-helical sheets into nanotubes (Kreutzberger et al., 2022). Interestingly, the cross-α configuration was also observed in the longer PSMβ2 variant, a 44-residue peptide that forms a helix-turn-helix motif, suggesting an additional mode of assembly based on mated helical sheets (Kreutzberger et al., 2022).

The remarkable variation in secondary structure observed between the cross-α fibrils of PSMα3 and PSMβ2, and the cross-β fibrils commonly found in human amyloids, highlights the presence of structurally-encoded functional specificity. Notably, this distinction becomes more intriguing when considering that closely related family members, PSMα1 and PSMα4, form the canonical cross-β amyloid fibrils (Salinas et al., 2018). While high-resolution structures of PSMα1 and PSMα4 are not available, their X-ray fiber diffraction pattern exhibits the characteristic cross-β reflections, and the crystal structures of their amyloidogenic segments reveal the tight assembly of parallel β-strands in a cross-β “steric-zipper” architecture (Salinas et al., 2018) (Figure 1). Exploiting the distinct fibril polymorphism observed between PSMα1’s cross-β and PSMα3’s cross-α structures, despite their homologous sequences originating from the same *S. aureus* operon, we aimed to investigate the relationship between activity, structure, and thermostability.

## Results

### Thermostability of PSMα1 and PSMα3 fibrils

**FIGURE 2:**
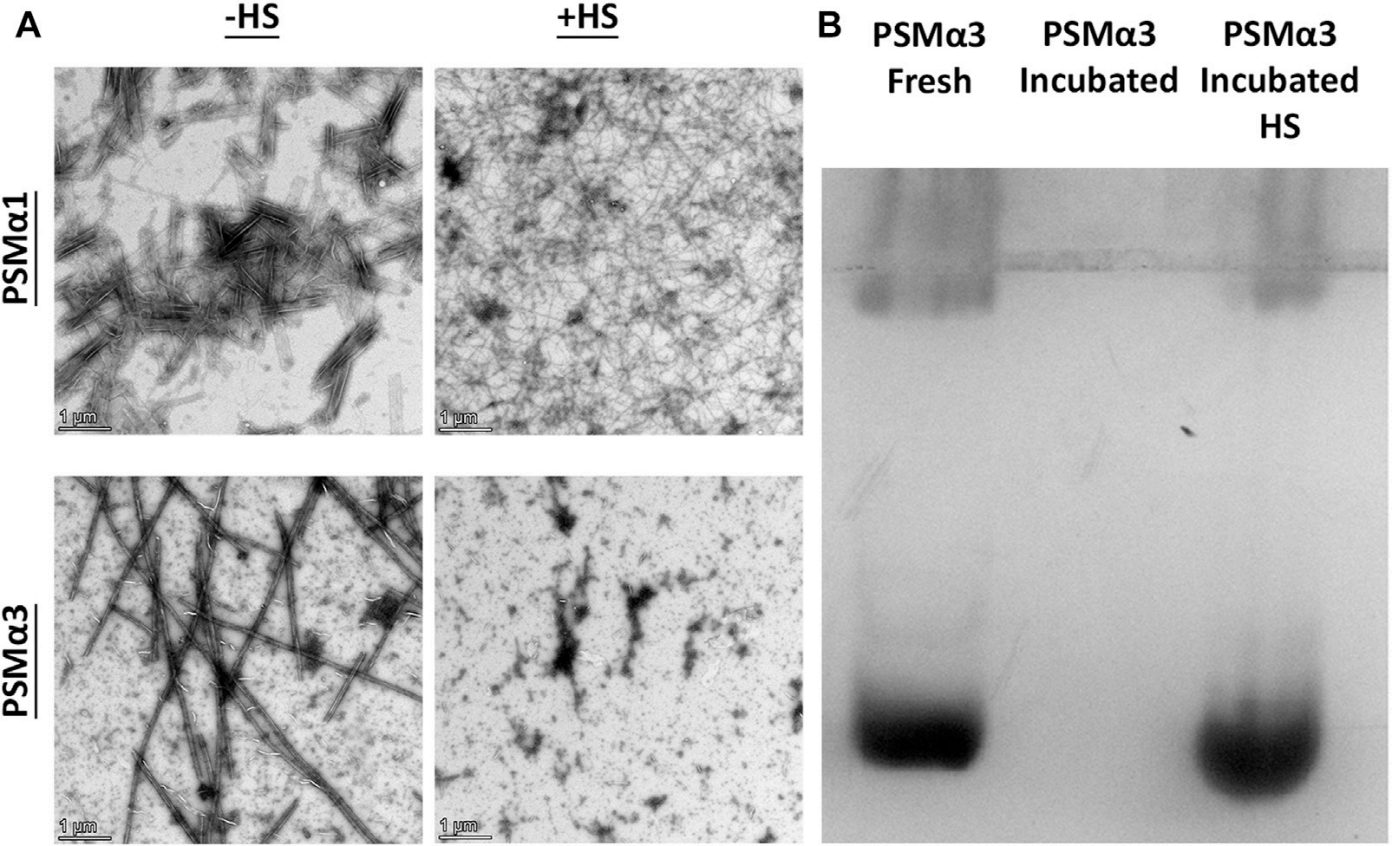
Thermostability of PSMα1 and PSMα3 Fibrils. **(A)** Transmission electron micrographs of PSMα1 and PSMα3 fibrils before and after heat shock. Scale bars represent 1 μm. **(B)** Native cationic gel electrophoresis stained with Coomassie brilliant blue. The lanes show PSMα3 in its freshly dissolved state, after incubation for 2 days to form fibrils, and after heat shock.

To assess the thermostability of PSMα1 and PSMα3 fibrils, we conducted heat shock (HS) experiments followed by visualization using transmission electron microscopy (TEM) (Figure 2A). After incubation at 37°C for 2 days, both PSMα1 and PSMα3 fibrils were subjected to HS at 80°C for 10 min. TEM micrographs revealed the presence of abundant PSMα1 fibrils before and after HS, indicating their resistance to heat. However, the HS-treated PSMα1 fibrils appeared thinner and denser, suggesting a potential influence on fibrillation rate and morphology. In contrast, TEM images of PSMα3 fibrils indicated their lack of thermostability, as the fibrils had disaggregated following HS treatment.

To further confirm the HS-induced disassembly of PSMα3 fibrils, we analyzed the mobility of PSMα3 using a cationic native gel (Figure 2B). Initially, freshly dissolved PSMα3 exhibited a band corresponding to soluble species that migrated on the gel. After incubation, PSMα3 fibrils became immobile on the gel, indicating their aggregated state. However, HS treatment resulted in a band similar to that of freshly dissolved PSMα3, providing additional evidence for the disaggregation of PSMα3 fibrils back into soluble species.

Overall, our findings demonstrate the contrasting thermostability of PSMα1 and PSMα3 fibrils, with PSMα1 exhibiting resistance to HS-induced changes in structure and PSMα3 undergoing disaggregation upon heat shock treatment.

### Size distribution of PSMα1 and PSMα3 before and after heat shock

**FIGURE 3:**
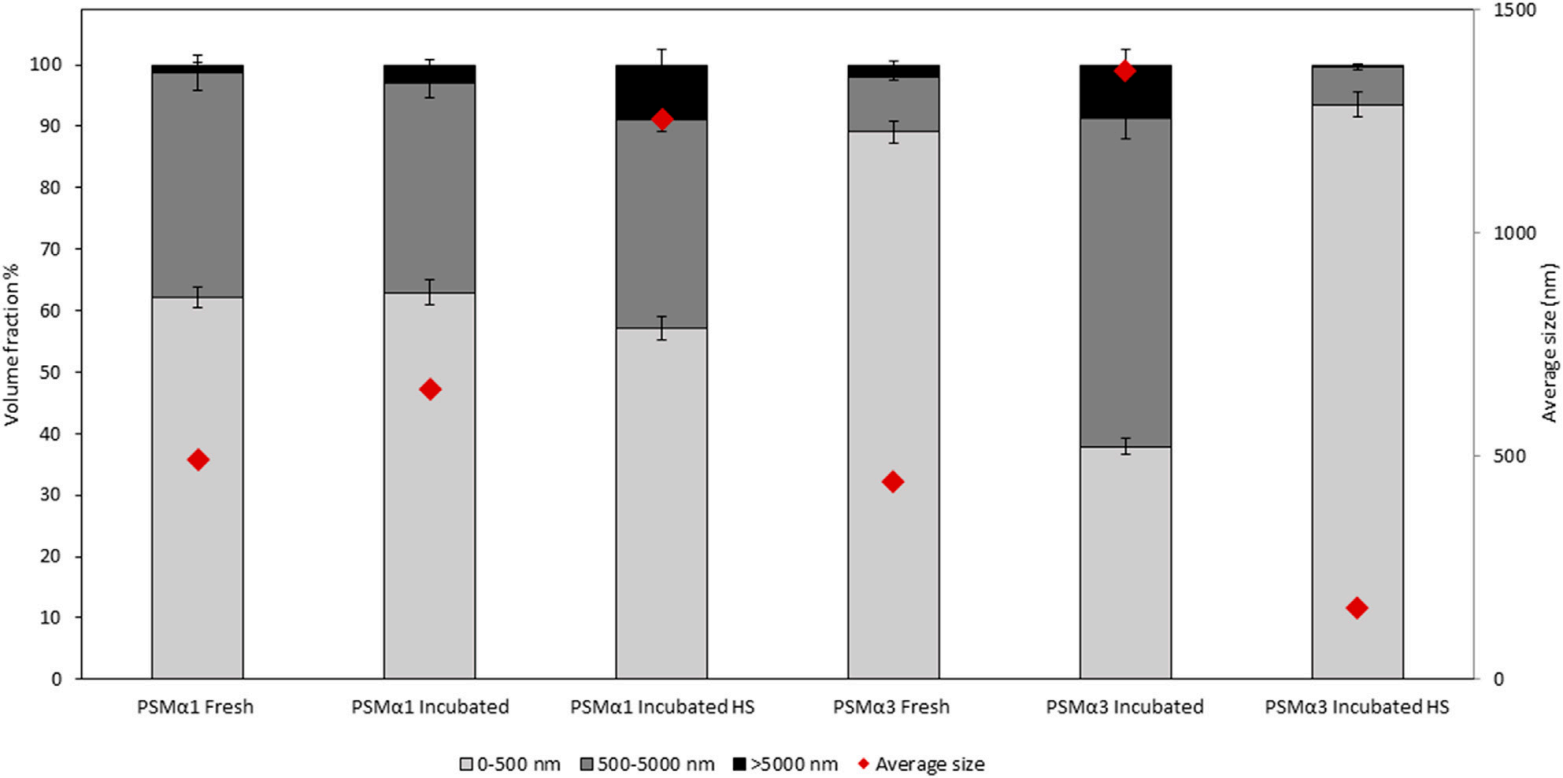
Particle size and volume distribution of PSMα1 and PSMα3. The particle size distribution of PSMα1 and PSMα3 was analyzed using dynamic light scattering (DLS) in various states: freshly dissolved, after 2-day incubation, and before and after heat shock. The particles were categorized into three size ranges: <500 nm, 500–5,000 nm, and >5,000 nm. The volume distribution of each size population was represented using different shades, and error bars were included. The average size of the total particle populations in each sample was indicated by red rhombuses.

The size distribution of PSMα1 and PSMα3 was determined using dynamic light scattering (DLS) in different states: freshly dissolved, incubated before and after heat shock. The particle size distribution was plotted against volume, dividing the particles into three size ranges: <500 nm, 500–5,000 nm, and >5,000 nm (Figure 3; Table 1).

**TABLE 1:** Particle size and volume distribution of PSMα1 and PSMα3.

Peptide	HS	Subpopulation	Average diameter (mean ± S.E, nm)	Volume distribution (mean ± S.E, %)	Total particles average diameter (mean ± S.E, nm)
PSMα1 Fresh	−	0–500 nm	85.9 ± 9.1	62.1 ± 1.7	492.3 ± 90.8
		500–5,000 nm	996.9 ± 68.7	36.6 ± 2.8	
		>5,000 nm	5,732 ± 13	1.3 ± 0.4	
PSMα1 Incubated	−	0–500 nm	143.6 ± 15.4	63 ± 2	651.3 ± 123
		500–5,000 nm	1,149.7 ± 78.1	34.1 ± 2.3	
		>5,000 nm	5,728.8 ± 29.6	2.9 ± 0.8	
	+	0–500 nm	157.4 ± 16.1	57.1 ± 1.9	1,254.3 ± 353.8
		500–5,000 nm	1911.3 ± 198.3	33.9 ± 1.8	
		>5,000 nm	5,724.3 ± 139.4	9 ± 2.5	
PSMα3 Fresh	−	0–500 nm	109.9 ± 17.7	89.1 ± 1.8	443.8 ± 135.4
		500–5,000 nm	2,618.2 ± 89.2	8.9 ± 0.4	
		>5,000 nm	5,706.9 ± 28.7	2 ± 0.6	
PSMα3 Incubated	−	0–500 nm	170.8 ± 19.7	37.9 ± 1.4	1,361.6 ± 356.5
		500–5,000 nm	1,487.1 ± 224.3	53.3 ± 3.3	
		>5,000 nm	5,729.1 ± 112.5	8.8 ± 2.4	
	+	0–500 nm	76.3 ± 14.8	93.5 ± 2	159.8 ± 41
		500–5,000 nm	1,115.3 ± 21.9	6.2 ± 0.4	
		>5,000 nm	5,720.5 ± 4.4	0.3 ± 0.1	

The table presents the DLS measurement results for particle size and volume distribution of PSMα1 and PSMα3, as depicted in Figure 3.

For PSMα1, the size population distribution remained relatively stable before and after incubation, with a slight increase in the average particle size from approximately 492 nm–651 nm. The percentage of large particles (>5,000 nm) also increased, which correlates with the formation of mature fibrils observed in the electron micrographs (Figure 2), although they probably represent a small fraction of the population of species. After HS, there was a further increase in the percentage of large particles at the expense of small particles, leading to an overall average particle size of approximately 1,254 nm. These results suggest that PSMα1 fibrillation is temperature-driven and indicate the thermostability of the fibrils.

In contrast, PSMα3 exhibited a distinct behavior compared to PSMα1. Incubation caused a substantial increase in the average particle size from approximately 444 nm–1,362 nm for PSMα3, accompanied by a decrease in the percentage of small-sized particles (<500 nm) from 89% to 38%. However, following HS, there was a notable shift towards smaller particles, with particles smaller than 500 nm constituting approximately 93.5% of the population. The total average size decreased to 160 nm, the smallest among all tested samples. These findings align with the TEM images and native gel results, which demonstrate the HS-induced breakdown of PSMα3 fibrils (Figure 2).

These results provide comprehensive information on the size distribution of PSMα1 and PSMα3 fibrils and confirm the thermostability of PSMα1 fibrils while demonstrating the sensitivity of PSMα3 fibrils to heat shock.

### Heat shock induces distinct alterations in the secondary structure of PSMα1 and PSMα3 fibrils

**FIGURE 4:**
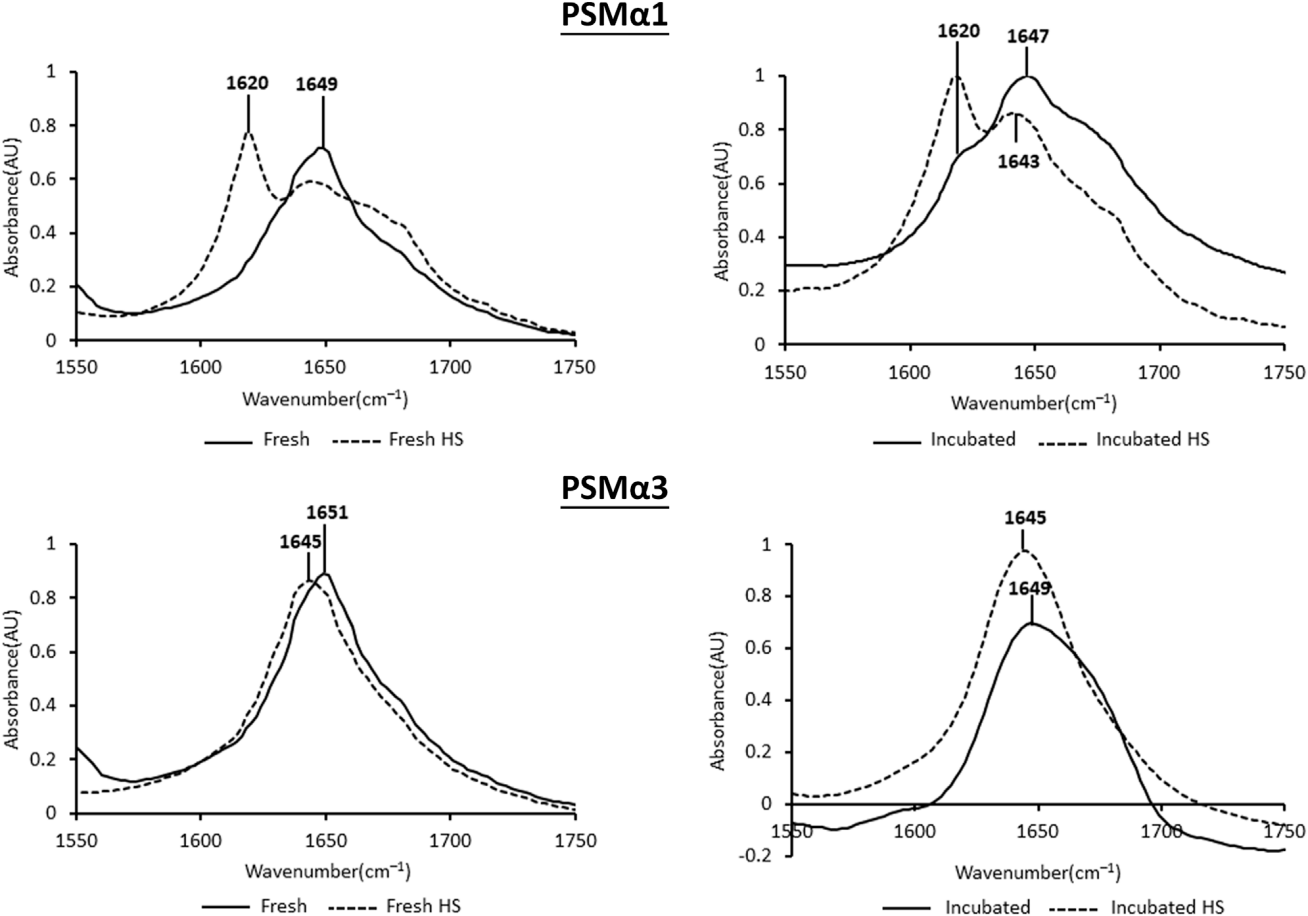
Secondary structure of PSMα1 and PSMα3. The figure displays the attenuated total internal reflection Fourier transform infrared (ATR-FTIR) spectra of the amide I′ region for PSMα1 and PSMα3 fibrils. The spectra are shown for samples incubated for 2 h or 2 days, both before and after heat shock. The peaks in the spectra were identified using the second derivative calculated with OPUS software.

Heat shock treatment has distinct effects on the secondary structure of PSMα1 and PSMα3 fibrils, as determined by attenuated total internal reflection Fourier transform infrared (ATR-FTIR) spectroscopy (Figure 4). The secondary derivative of each FTIR spectrum was calculated to identify the major peaks contributing to the overlapping signals. Peaks in the regions of 1,611–1,630 cm^−1^ and ∼1,685–1,695 cm^−1^ indicate rigid cross-β fibrils, while peaks in the region of 1,637–1,645 cm^−1^ represent disordered species (Goormaghtigh et al., 1990; Zandomeneghi et al., 2004; Sarroukh et al., 2013; Moran and Zanni, 2014). Additionally, peaks in the region of 1,646–1,662 cm^−1^ suggest the presence of α-helices, but may overlap with random coil structures, especially for broad peaks (Barth and Zscherp, 2002; Cracchiolo et al., 2022).

PSMα1, after 2 h of incubation, exhibited a dominant helical population with a peak at 1,649 cm^−1^. After 2 days of incubation, a minor cross-β population emerged alongside the major random/α-helical population. Heat shock treatment had a significant impact on both the 2-h and 2-day incubated samples, leading to a structural transition towards a predominantly cross-β conformation with a peak at 1,620 cm^−1^ (Figure 4).

PSMα3, after 2 h or 2 days of incubation, displayed peaks at 1,651 cm^−1^ and 1,649 cm^−1^, respectively, indicating a predominant helical population, as previously shown (Tayeb-Fligelman et al., 2020; Zaman and Andreasen, 2020). In contrast to PSMα1, heat shock induced a changed into disordered morphology in both PSMα3 samples, with a peak at 1,645 cm^−1^ (Figure 4). These findings are consistent with the results obtained from DLS (Figure 3), TEM, and native gel analyses (Figure 2), suggesting that heat shock disaggregates PSMα3 α-helical fibrils into soluble and less ordered species.

### Recovery of PSMα3 fibrils following heat shock

**FIGURE 5:**
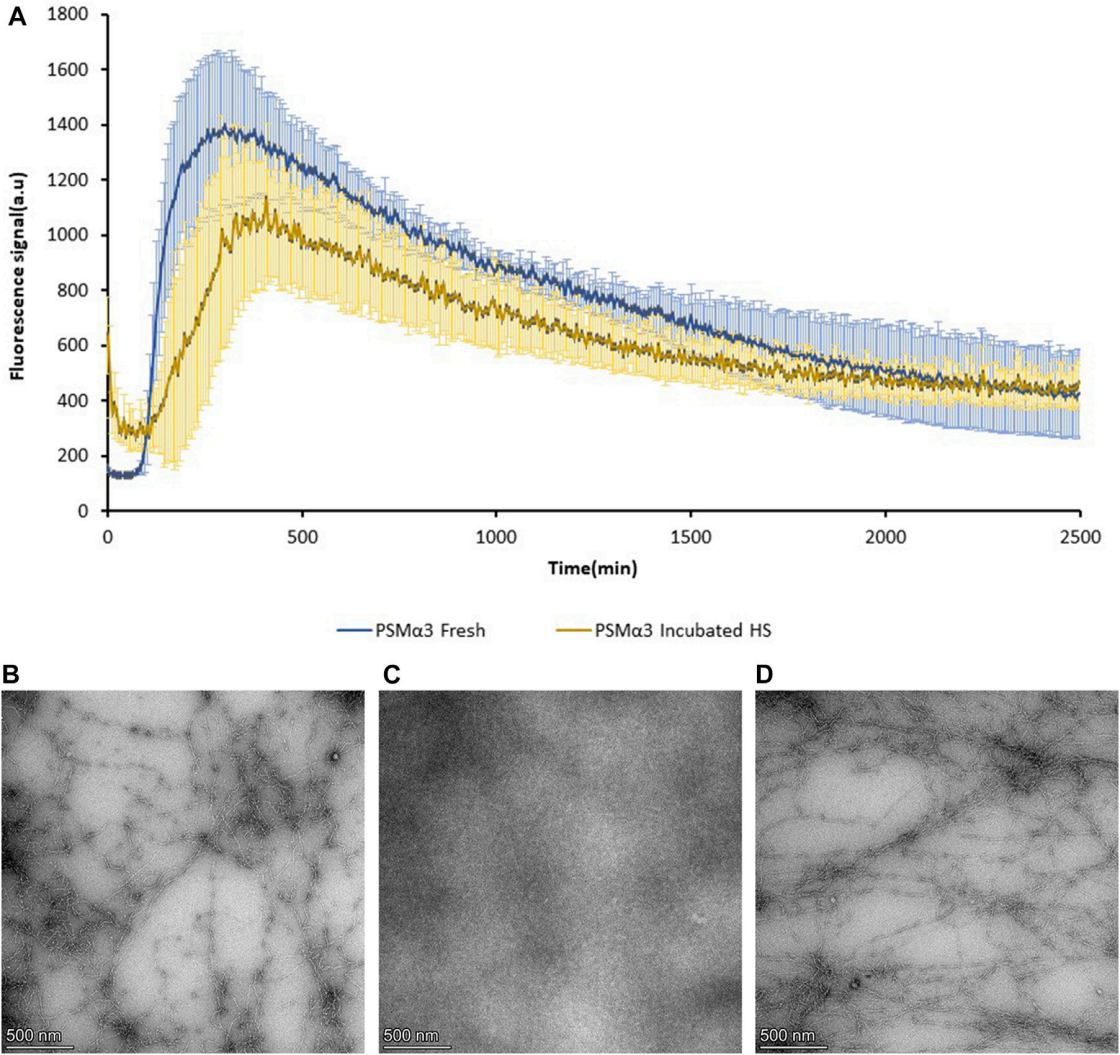
Reversibility of PSMα3 fibril formation following heat shock. **(A)** Fibrillation kinetics of 100 µM freshly dissolved PSMα3 and 2-day incubated PSMα3 after heat shock, monitored by Thioflavin-T (ThT) binding. The graph represents the mean fluorescence readings of triplicate ThT measurements. Error bars indicate standard errors of the mean. **(B–D)** Transmission electron microscopy (TEM) micrographs of PSMα3 incubated for 2 days before heat shock **(B)**, after heat shock **(C)**, and following further incubation for 2 days **(D)**. All scale bars represent 500 nm.

To assess the recovery of PSMα3 fibril formation after heat shock-induced disaggregation, thioflavin-T (ThT) fluorescence kinetics and TEM visualization were employed (Figure 5). Freshly dissolved PSMα3 displayed the characteristic amyloid lag time followed by rapid aggregation, consistent with previous findings (Tayeb-Fligelman et al., 2020; Zaman and Andreasen, 2020). After incubation and heat shock, PSMα3 exhibited a similar fibrillation curve, albeit with a slightly longer lag time. TEM micrographs confirmed the initial formation of PSMα3 fibrils (Figure 5B), the heat shock-induced disaggregation (Figure 5C), and subsequent reformation of PSMα3 fibrils with a similar morphology (Figure 5D). These results indicate the reversible nature of fibril formation following heat stress.

### Effect of heat shock on the cytotoxicity of PSMα1 and PSMα3

**FIGURE 6:**
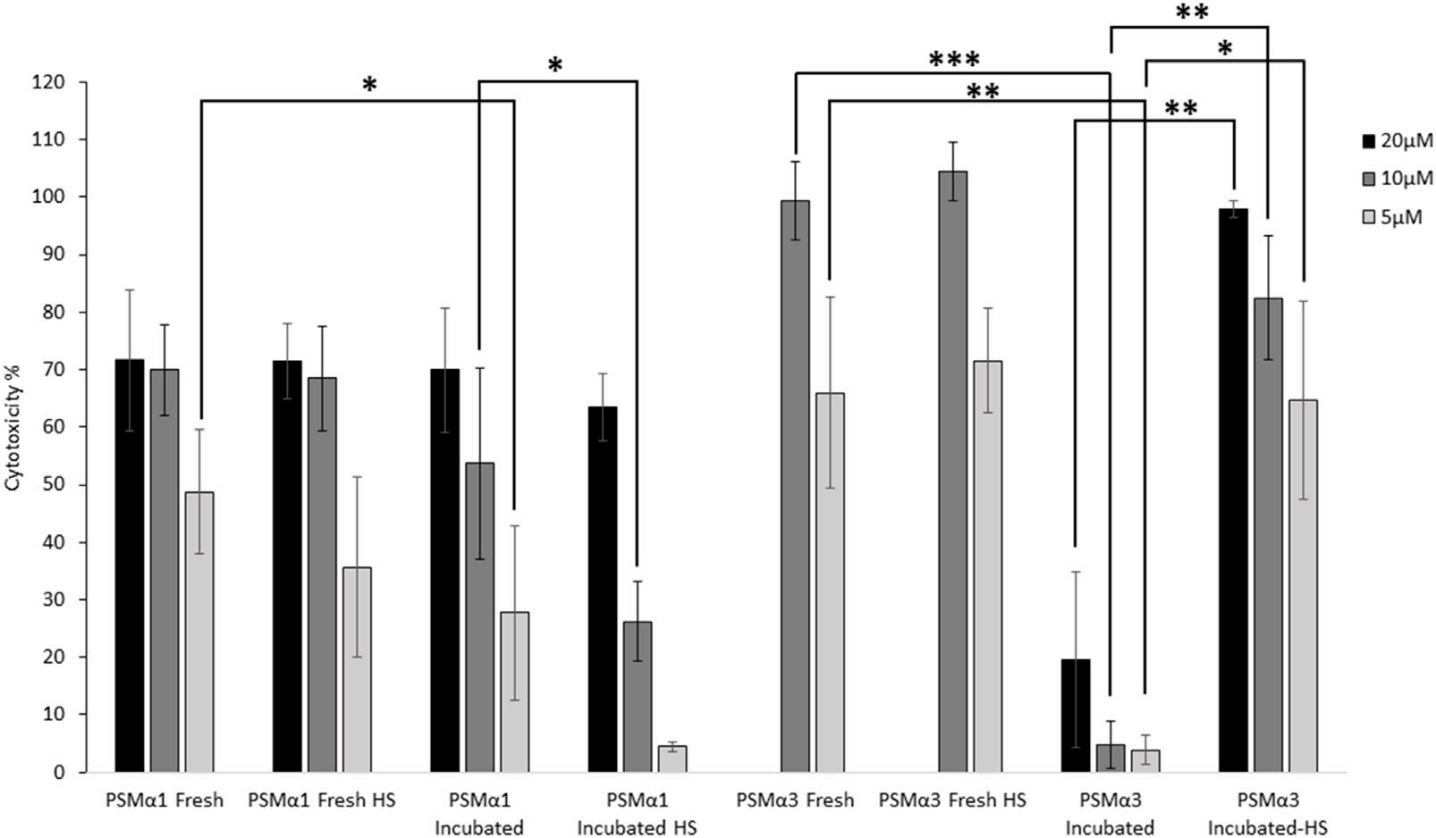
Cytotoxicity of PSMα1 and PSMα3. Cytotoxicity against T cells of both freshly dissolved and incubated PSMα1 and PSMα3, before and after heat shock, was assessed using the lactate dehydrogenase colorimetric assay (LDH). The experiment was done in at least three replicates and repeated three times on different days to ensure the reliability of the results. The percentage of cytotoxicity for each sample was calculated by averaging the data from all repetitions, and error bars represent the standard errors of the mean. We used statistical tests to determine the significance of the differences in cytotoxicity levels between the freshly dissolved peptide and the incubated sample at each concentration, as well as between the incubated samples before and after heat shock. A single asterisk (*) was used to denote a *p*-value between 0.01 and 0.05, indicating a significant difference. Two asterisks (**) were used for *p*-values between 0.01 and 0.001, indicating a more significant difference. Three asterisks (***) were used for *p*-values between 0.0001 and 0.001, indicating a highly significant difference. We only displayed differences that were significant and could be attributed to the incubation or heat shock process.

The cytotoxicity of PSMα1 and PSMα3 against T2 cells was assessed by measuring lactate dehydrogenase (LDH) release. The cytotoxicity was evaluated for freshly dissolved peptides, peptides incubated for 2 days, and incubated fibrils before and after heat shock (Figure 6).

When freshly dissolved, both PSMα1 and PSMα3 exhibited high toxicity, with 10 μM concentration resulting in 70% and 100% cell death, respectively. Heat shock of the freshly dissolved samples did not significantly alter their toxicity, indicating the stability of the peptides at 80°C. After 2 days of incubation, PSMα1 showed a slight reduction in cytotoxicity. However, heat shock of the incubated samples further decreased the cytotoxicity of PSMα1. At a concentration of 5 μM, cell death reduced to only 4% after heat shock, compared to 49% for the fresh sample. At 10 μM, cytotoxicity decreased to 26% (compared to 70%). In contrast, PSMα3 cytotoxicity was abolished after 2 days of incubation, but almost fully restored after heat shock (Figure 6).

## Discussion

The variation in thermostability among different functional amyloids, including PSMαs, is likely attributed to their distinct architectures, which are determined by their biological functions in diverse environments. Currently, our knowledge regarding the thermostability of functional amyloids is limited. However, studies on human proteins capable of forming amyloids, such as lysozyme, have revealed that the thermostability of fibrils is influenced by various factors, including pH, ionic strength, and the presence of ligands or other molecules (Pace and McGrath, 1980; James and McManus, 2012; Blumlein and McManus, 2013; Venkataramani et al., 2013). Previous investigations have indicated that PSMα1 and PSMα4 exhibit thermostability, maintaining their β-sheet structure even at high temperatures of up to 95°C. On the other hand, PSMα3 fibrils demonstrate structural instability when exposed to temperatures above 50°C (Zaman and Andreasen, 2020). Furthermore, differences in thermostability have been observed at the level of amyloid segments. For instance, the PSMα4 segment IIKIIK, which forms a ‘steric-zipper’ cross-β crystal structure, exhibits thermostability. Conversely, the PSMα3 segment LFKFFK, which adopts a unique amyloid-like structure with out-of-register β-sheets, lacks thermostability. These findings suggest a correlation between the atomic structure of the fibril and its ability to withstand high temperatures (Salinas et al., 2018).

Although amyloid fibrils are typically characterized by tightly paired sheets, they exhibit high levels of polymorphism, even within the same sequence (Cao et al., 2019; Kollmer et al., 2019). This polymorphic nature of amyloids deviates from Anfinsen’s “one-sequence-one-structure” rule (Anfinsen, 1973; Li and Liu, 2020). An intriguing example of extreme polymorphism is found in the antimicrobial peptide uperin 3.5, which is secreted by Uperoleia mjobergii (Mjoberg’s toadlet). Uperin 3.5 has been shown to adopt two distinct secondary structures, namely, cross-α and cross-β fibrils, as evidenced by high-resolution structures and biophysical measurements (Salinas et al., 2021; Bücker et al., 2022). Uperin 3.5 fibrils share similarities with PSMα1, as they exhibit thermostability at 60°C, accompanied by an increase in β-rich content upon exposure to heat, and reduced cytotoxicity (Salinas et al., 2021). This ability to switch to more stable β-rich fibrils under the influence of time and temperature grants uperin 3.5 its thermostability and diminishes its cytotoxicity. However, in the presence of membrane lipids, uperin 3.5 can partially revert back to cross-α fibrils, thereby retaining its antimicrobial activity (Salinas et al., 2021). These observations emphasize the intricate and dynamic characteristics of amyloid structures and the potential functional consequences associated with their versatility.

In contrast to globular proteins, where specific secondary structures may be influenced by the overall tertiary context rather than inherent preferences (Minor and Kim, 1994; Kolodny et al., 2021; Qiu et al., 2022), amyloids exhibit a substitution of compact globular folds with the tight packing of self-assembling molecules, which affects local structure preferences. The ability of chameleon proteins to adopt diverse secondary structures in response to external stimuli has been observed, including in various nanofibers such as cross-α/β structures (Kabsch and Sander, 1984; Minor and Kim, 1994; Zhang and Rich, 1997; Magzoub et al., 2002; Andreola et al., 2003; Hamodrakas et al., 2007; El Amri and Nicolas, 2008; Louros et al., 2015; Kim et al., 2016; Roberts et al., 2018; Mondal et al., 2019; Doti et al., 2020; Hema and Sureshan, 2020; Ragonis-Bachar et al., 2022). In human amyloids associated with neurodegeneration and amyloidosis, the transition from unstructured or α-helical conformations or intermediates to cross-β fibrils is a well-studied example of a secondary structure switch (Kelly, 1996; Groß, 2000; Kim et al., 2016). While prefibrillar oligomeric conformations have been implicated in the pathogenesis of amyloid-related diseases, it is worth noting that some of these soluble species retain α-helical structures (Ghosh et al., 2015; Jiang et al., 2019; Yang et al., 2020).

Functional amyloid secondary structure switches likely evolved as a regulatory mechanism, influenced by environmental conditions, possibly serving to switch between storage and toxicity states (Salinas et al., 2021; Bücker et al., 2022; Ragonis-Bachar et al., 2022). It has been observed that cytotoxicity against human cells is associated with an inherent or lipid-induced α-helical fibril structure (Ragonis-Bachar et al., 2022). In our study, we demonstrate that the cytotoxicity of PSMα1 and PSMα3 is reduced after a 2-day incubation period (Figure 6), possibly due to a decrease in the effective concentration of soluble species required to interact with the cell membrane. PSMα3 exhibited a more significant loss of activity, which may correlate with its faster fibrillation rate compared to PSMα1 (Zaman and Andreasen, 2020). While incubated PSMα3 fully recovered its activity after heat shock, likely due to the restoration of a similar concentration of soluble species, PSMα1 displayed even further reduced activity due to HS-induced fibrillation into cross-β fibrils (Figures 2–4).

The lack of thermostability in PSMα3 suggests a requirement for reversibility of activity, which is enabled by the formation of cross-α rather than cross-β fibrils. Consistent with this, previous studies have shown that cross-α fibrillation enhances PSMα3 cytotoxicity against human T2-cells (Tayeb-Fligelman et al., 2017; 2020; Malishev et al., 2018). We hypothesized that PSMα3 co-aggregates with human cell membranes in a process that relies on the continuous presence of monomers and the ability to form cross-α fibrils, implying that toxicity is a complex process rather than being attributed to a specific toxic entity (Tayeb-Fligelman et al., 2020). In contrast to PSMα3, the cross-β fibrillation of PSMα1, which is further facilitated by heat (Figures 2–4), leads to an irreversible loss of toxic activity (Figure 6). This aligns with the role of PSMα1 in biofilm structuring, which necessitates the formation of highly stable and robust fibrils. Similarly, human amyloids associated with fatal diseases and biofilm-associated curli fibrils are known for their exceptional mechanical and chemical stability (Knowles et al., 2007; DePas and Chapman, 2012).

Overall, our findings demonstrate a correlation between PSMα1&3 fibril morphology, secondary structure switching, thermostability, activity, and regulation by environmental components. These insights pave the way for the design of innovative active peptides that can be controlled by specific cues.

## Methods

### Peptide preparation and heat shock treatment

PSMα1 and PSMα3 (>98% purity) were purchased from GL Biochem (Shanghai) Ltd. The peptides were dissolved at 5 mM in 20% dimethyl sulfoxide (DMSO) and 80% double-distilled water (DDW) and incubated at 37°C for 2 h or 2 days. Heat shock treatment was induced by incubating the samples at 80°C for 10 min.

### Lactate dehydrogenase (LDH) release cytotoxicity assay

The cytotoxicity assay was performed using human lymphoblast T2 cells (174 x CEM.T2) obtained from ATCC® (CRL-1992™). The cells were cultured in the assay medium, namely, RPMI 1640 medium with L-glutamine from Sigma (Israel) and supplemented with penicillin (100 U/mL), streptomycin (0.1 mg/mL), and 10% heat-inactivated fetal calf serum from Biological Industries (Israel). The cells were maintained at 37°C in a 5% CO2 environment. For the cytotoxicity assay, freshly dissolved peptides and samples incubated for 2 days at 37°C, with and without subsequent heat shock treatment, were used. The peptide samples were diluted to a concentration of 1 mM in DDW and then further diluted to 160 μM in the assay medium.

Serial twofold dilutions of the peptides were prepared in the assay medium, and 50 μL of each dilution was added in triplicate to a 96-well plate. The plate was incubated for 15 min at room temperature. The T2 cells were washed and resuspended in the assay medium to a concentration of 0.15 × 106 cells/mL, and 50 μL of the cell suspension was added to the diluted peptides in the plate. The plate was then incubated for 30 min at 37°C in a 5% CO_2_ environment. Serial twofold dilutions of the peptides were prepared in the assay medium, and 50 μL of each dilution was added in triplicate to a 96-well plate. The plate was incubated for 15 min at room temperature. The T2 cells were washed and resuspended in the assay medium to a concentration of 0.15 × 106 cells/mL, and 50 μL of the cell suspension was added to the diluted peptides in the plate. The plate was then incubated for 30 min at 37°C in a 5% CO_2_ environment.

The quantification of cell lysis was performed using the lactate dehydrogenase (LDH) release assay according to the manufacturer’s instructions. The LDH release was measured using the LDH Cytotoxicity Detection Kit Plus from Roche Applied Science (Germany), including all recommended controls. The absorbance at 490 nm and 690 nm was measured using a plate reader (FLUOstar Omega, BMG Labtech, Germany). The absorbance values at 690 nm were subtracted from the absorbance values at 490 nm, and the average absorbance values of triplicate samples and controls were calculated after subtracting the background. The experiment was repeated three times on different days. The percentage of cytotoxicity for each sample was determined by averaging the data from all replicates and repeats. Error bars were included to represent the standard errors of the mean, providing a measure of the variability in the results. The *p*-value was calculated using a *t*-test to compare the % cytotoxicity of the freshly dissolved peptide with the incubated peptide, and of the incubated samples before and after heat shock, at each concentration, as displayed in Figure 6.

### Thioflavin T fluorescence fibrillation kinetics assay

ThT is a widely used “gold standard” stain for identifying and studying the kinetics of amyloid fibril formation, both *in vivo* and *in vitro*. Fibrillation curves in the presence of ThT typically show a lag time for the nucleation step, followed by rapid aggregation. To ensure the presentation of the fibrillation lag time, PSMα3 was pre-treated prior to the experiment by dissolving in 1:1 1,1,1,3,3,3-Hexafluoroisopropanol (HFIP) and Trifluoroacetic acid (TFA) to a concentration of 1 mg/mL, followed by 10 min bath-sonication, at room temperature. The organic solvent was evaporated using a mini-rotational vacuum concentrator (Christ, Germany) at 1,000 rpm for 2 h, at room temperature. For the experiment, fresh PSMα3 peptides and incubated PSMα3 after HS were diluted to 100 μM in phosphate buffer, pH 7.5, containing filtered ThT diluted from the stock prepared in UPddw. The final concentrations for each reaction were 100 µM peptide and 200 µM ThT. Blank solutions containing all components except from the peptides/fibrils were also prepared for each reaction. The reaction was carried out in black 96-well flat-bottom plates (Greiner bio-one) covered with a thermal seal film (EXCEL scientific) and incubated in a plate reader (OMEGA) at a temperature of 37°C with shaking at 500 rpm for 85 s before each reading cycle, and up to 1,000 cycles of 6 min each. Measurements were performed in triplicate. Fluorescence was measured by excitation at 438 ± 20 nm and emission at 490 ± 20 nm over a period of approximately 100 h. All triplicate values were averaged, appropriate blanks were subtracted, and the resulting values were plotted against time. Calculated standard errors of the mean are shown as error bars. The whole experiment was repeated at least three times on different days.

### Transmission electron microscopy (TEM)

Samples (4–5 µL) of incubated PSMα1 and PSMα3, with and without HS treatment were applied directly onto glow-discharged (easiGlow; Pelco, Clovis, CA, United States, 15 mA current; negative charge; 25 s time) 400 mesh copper grids, with a grid hole size of 42 μm, stabilized with Formvar/carbon (Ted Pella, Inc.). Samples were allowed to adhere for 60 s. Samples were then stained with 1% uranyl acetate solution (Electron Microscopy Science, 22400-1) for 60 s. Samples were examined in the Ilse Katz Institute for Nanoscale Science and Technology, Ben Gurion University of the Negev, Israel, with a ThermoFisher Scientific (FEI) Talos F200C transmission electron microscope operating at 200 kV, equipped with a Ceta 16M CMOS camera.

### Attenuated total reflectance (ATR) fourier transform infrared (FTIR) spectroscopy

PSMα1 and PSMα3 were subjected to HFIP pretreatment according to the method described for the ThT experiment. The lyophilized peptides were then dissolved in 5 mM hydrochloric acid (HCl) at a concentration of 1 mg/mL and sonicated for 5 min at room temperature using a bath sonicator. Subsequently, the peptide solution was frozen in liquid nitrogen and lyophilized overnight to ensure complete drying. This process was repeated twice to ensure the removal of any residual TFA, as TFA exhibits a strong FTIR signal in the amide I′ region of the spectra. Finally, the peptides were dissolved in deuterium oxide (D2O) at a concentration of 1 mg/mL, frozen in liquid nitrogen, and lyophilized overnight to achieve complete dryness. This double procedure was repeated. The dried peptides were then dissolved in D2O to a concentration of 5 mM, and samples were prepared for measurement after 48 h of incubation, both with and without heat shock.

A volume of 5 μL of each sample was spread onto the surface of the ATR module and allowed to dry. Absorption spectra were recorded using a Tensor 27 FTIR spectrometer (Bruker Optics) on the dried samples. Measurements were conducted in the wavelength range of 400–4,000 cm^−1^ with a step size of 2 cm^−1^, and an average of 32 scans was taken. Background (air) and blank (D2O) absorbances were measured and found to have a negligible signal. Peaks were selected by the instrument based on the second derivative using OPUS software.

### Dynamic light scattering (DLS)

PSMα1 and PSMα3 peptides were dissolved in a solution containing 20% DMSO and 80% DDW at a concentration of 5 mM. They were then incubated at 37°C for 2 and 48 h. The freshly dissolved peptides underwent pretreatment by dissolving them in HFIP at a concentration of 1 mg/mL, followed by a 10-min bath sonication at room temperature. The organic solvent was evaporated using a mini-rotational vacuum concentrator (Christ, Germany) at 1,000 rpm for 2 h at room temperature. The treated peptides were also dissolved in a solution containing 20% DMSO and 80% DDW at a concentration of 5 mM.

Freshly dissolved samples, and incubated ones with and without HS treatment (20–30 μL), were mounted using 1.0 × 1.0 mm disposable cuvette capillaries with a thickness of 200 μm (Malvern, ZSU0003), which were then sealed with clay. The capillaries were placed within a low-volume disposable sizing cell Kitholder (Malvern, ZSU1002). Light scattering readings were measured using a ZetaSizer Ultra (Ultra ZS; Malvern). Hydrodynamic radii (Rh) were determined using backscattered light at a fixed angle of 90°. A 633 nm wavelength He–Ne laser was used. The cell holder was maintained at 37°C throughout the measurement. The scattering data were obtained from two (fresh samples) or three (incubated samples) different measurements performed on different days, each consisting of nine and thirteen consecutive scans, respectively.

For each peptide, the particle size distribution was plotted against intensity or volume and each representation provides a different perspective. In particular, even for particles with a perfect spherical shape and smooth morphology, the intensity distribution for a fixed mean size shifts towards larger sizes with increasing polydispersity (Borah and Verbruggen, 2022). Therefore, we chose to represent the particle size distribution plotted against volume, to be able to better observe smaller particles. The mean volume intensities of the particles were divided into three subpopulations based on their size: 0–500 nm, 500–5,000 nm, and greater than 5,000 nm. To calculate the average size of particles in each subpopulation, the sum of the multifaction of different particles sizes in the corresponding percentage of volume intensity for each size was divided by the sum to the total percentage of volume intensity for the range. To calculate the error presented in Figure 3 and Table 1, the standard deviation was divided by 100, and the square root of the number of measurements.

To determine the average size of particles within each subpopulation presented in Figure 3 and Table 1, the following calculation was performed: the sum of the product of different particle sizes and their corresponding percentage of volume intensity was divided by the total percentage of volume intensity within the size range. To calculate the error, the standard deviation was divided by the square root of the number of measurements and by 100.

### Cationic native gel electrophoresis

Native gel was prepared as follows: The separating gel consisted of 385 mM acetate-KOH pH-4.3, 11% glycerol, 10% acrylamide 35.5:1, 0.12% ammonium persulfate (APS) and 0.15% tetramethylethylenediamine (TEMED). The stacking gel consisted of 62.5 mM acetate-KOH pH = 6.8, 8% acrylamide 35.5:1, 0.1% APS and 0.1% TEMED. The separating gel solution was dispensed into the gel template, covered with isopropanol and incubated for polymerization for 1 h at room temperature. After polymerization, residual isopropanol was removed and stacking gel was added for 1 h to allow complete polymerization on top of the separating gel. The running buffer contained 0.35 M β-alanine and 0.14 M acetate at pH 4.3. PSMα3 samples of freshly dissolved peptide and after incubation, with and without HS treatment, were loaded in X5 sample buffer containing 37.2% glycerol, 62.5 mM acetate KOH pH 6.8% and 0.01% methyl green. The gel was run on Bio-Rad mini-PROTEAN tetra system units with reverse polarity at a constant voltage of 150 V.

## Data Availability

The original contributions presented in the study are included in the article/Supplementary material, further inquiries can be directed to the corresponding author.
